# Influence of cutting parameters on the depth of subsurface deformed layer in nano-cutting process of single crystal copper

**DOI:** 10.1186/s11671-015-1082-1

**Published:** 2015-10-09

**Authors:** Quanlong Wang, Qingshun Bai, Jiaxuan Chen, Hao Su, Zhiguo Wang, Wenkun Xie

**Affiliations:** School of Mechatronics Engineering, Harbin Institute of Technology, Harbin, 150001 People’s Republic of China; Center for Precision Engineering, Harbin Institute of Technology, Harbin, 150001 People’s Republic of China; Beijing Institute of Aerospace Control Device, Beijing, 100039 People’s Republic of China

**Keywords:** Nano-cutting, Molecular dynamics, Subsurface deformed layers, Cutting parameters

## Abstract

Large-scale molecular dynamics simulation is performed to study the nano-cutting process of single crystal copper realized by single-point diamond cutting tool in this paper. The centro-symmetry parameter is adopted to characterize the subsurface deformed layers and the distribution and evolution of the subsurface defect structures. Three-dimensional visualization and measurement technology are used to measure the depth of the subsurface deformed layers. The influence of cutting speed, cutting depth, cutting direction, and crystallographic orientation on the depth of subsurface deformed layers is systematically investigated. The results show that a lot of defect structures are formed in the subsurface of workpiece during nano-cutting process, for instance, stair-rod dislocations, stacking fault tetrahedron, atomic clusters, vacancy defects, point defects. In the process of nano-cutting, the depth of subsurface deformed layers increases with the cutting distance at the beginning, then decreases at stable cutting process, and basically remains unchanged when the cutting distance reaches up to 24 nm. The depth of subsurface deformed layers decreases with the increase in cutting speed between 50 and 300 m/s. The depth of subsurface deformed layer increases with cutting depth, proportionally, and basically remains unchanged when the cutting depth reaches over 6 nm.

## Background

In nano-cutting, the subsurface deformed (SSD) layers have great influence on machining dimensional accuracy, surface shape accuracy, and surface roughness, which even affect the mechanical performance and lifetime of machined components. The SSD layers are basically caused by the following three factors: foreign body embedment caused by chemical or physical adsorption [1], stress injury caused by residual stress [2], and the variation of material local crystal structure [3]. The depth of SSD layers is a vital parameter on predicating the quality of SSD layers, the value of which has an important effect on the subsequent technical machining, mechanical property, and lifetime of machined component. The subsurface damage layer in nano-cutting is so difficult to observe that it cannot be verified by using experimental method. However, the atomistic computer simulation provides an effective and promising method to examine the subsurface defect and measure the depth of SSD layers.

Based on the molecular dynamics simulation, a large number of scholars do a lot of research on nano-cutting process. Shimada and Ikawa et al. [4–6] performed molecular dynamics (MD) simulation of micro-cutting of free machining materials under perfect motion of a machine tool. Based on the radial distribution function, they found that the ultimate depth of the deformed layer of a specimen is 5.0 nm. Luo [7] demonstrated the shape transferability by using nanoscale multi-tip diamond tools in the diamond turning for scale-up manufacturing of nanostructures. Based on the change of atomic potential energy, Zhang [8] realized the quantitative characterization of subsurface damage layer’s depth in nano-cutting process of a single crystal copper. Zhu [9] studied the deformation-induced formation mechanism of stacking fault tetrahedron occurring in the deformation of single crystal gold nanowires. Uezakia [10] designed a cutting tool to generate a localized compressive stress to suppress unnecessary plastic flow and to improve the surface integrity of workpiece, which is verified by the MD simulation. Guenole [11] investigated the plastic deformation of Si nanowires controlled by native interface defects and analyzed the inner stress influence on the yield strain. Ma [12] studied the plastic deformation of nanowires and analyzed the surface-induced structural transformation in the deformation process. They found two mechanisms involved in the deformation, twinning and detwinning, and stress-induced martensitic phase transformation. Zhao [13] preformed the nanoindentation process via the single-point diamond turning surface of single crystal copper. Fang [14] studied the nanometric cutting of germanium by MD simulation and discussed the phase transformation process during nano-cutting process. Wang [15] exploited the numerical experiments to study the evaporation and explosive boiling of ultra-thin liquid argon film on aluminum nanostructure substrate.

In this paper, a series of simulations on nano-cutting process of single crystal copper are implemented by using MD method. Theoretical analysis and investigation on the properties and depth of SSD layers in nano-cutting process will provide much information on the mechanisms of the plastic deformation in the workpiece material. Firstly, the centro-symmetry parameter (CSP) is adopted to characterize the distribution and evolution of the subsurface defect structure. Secondly, the measure method of the SSD layers’ depth is introduced and the depth of the SSD layer variation with the cutting distance is investigated. At last, the effect of the cutting parameter on the depth of the SSD layers is studied by information statistics. The research will give a distinct understanding for the formation of the subsurface deformed layers and the effect of cutting parameter on the evolution of the SSD layers, and underpin the scientific development of nano-cutting.

## Methods

### Simulation model

**Fig. 1:**
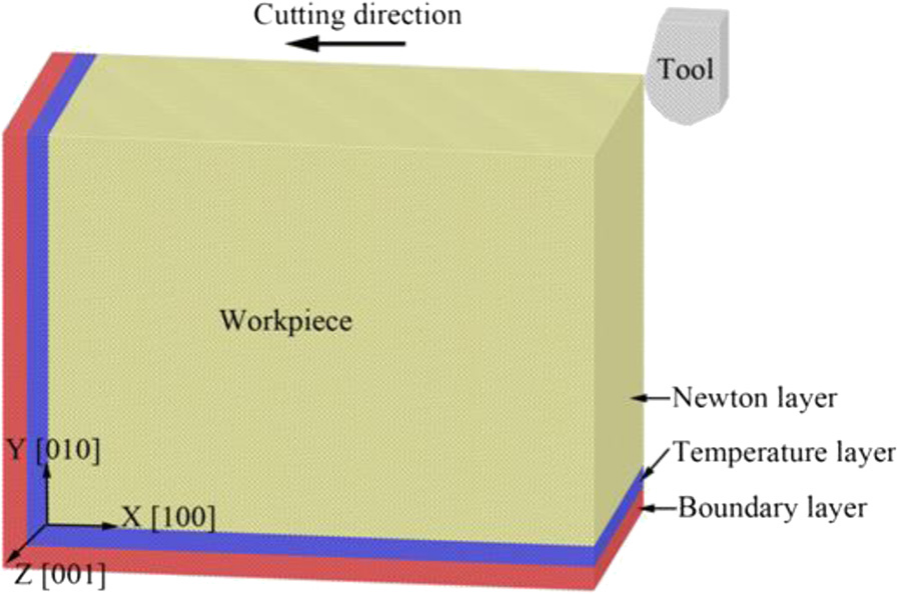
The MD simulation model in the nano-cutting

In this paper, the Large-scale Atomic/Molecular Massively Parallel Simulator is used to build the MD model and proceed with MD simulations of the nano-cutting process. The simulation model is shown as Fig. 1, which contains the monocrystalline copper workpiece and the diamond cutting tool. The workpiece is divided into a Newton layer, temperature layer, and boundary layer. The periodic boundary condition is applied along the Z direction of the workpiece to reduce the size effect. In order to avoid the interaction between carbon atoms and copper atoms, the cutting tool is put 4 nm top right of the workpiece at the initial state.

At the beginning of the simulation, a conjugate gradient method is used to carry out energy minimization for eliminating the initial unreasonable factors during modeling process. Then, the molecular dynamics relaxation is calculated for 100 ps by using the Nosé-Hoover thermostat method, which makes the system temperature up to 293 K. Thereafter, the cutting tool moves along Z [001] direction at a certain speed of 50 m/s to start the nano-cutting process.

In order to study the influence of the cutting depth on the depth of subsurface deformed layers, the cutting depth of the nano-cutting is changed from 1 to 10 nm. The maximum cutting distance is 40 nm. The other conditions and parameters related to the simulation are listed in Table 1.

**Table 1:** MD simulation conditions in 3D nano-machining

Machining parameters	Value
Potential function	Tersoff, Morse, EAM
Workpiece	Single crystal copper
Tool	Diamond
Lattice structure	FCC
Workpiece size	40 nm × 30 nm × 22 nm
Tool rake angle^*α*^	15°
Tool clearance angle^*β*^	8°
Tool edge radius *R*	3.0 nm
Cutting direction	(100) [100]
Cutting depth	1~10 nm
Cutting speed	50~300 m/s
Timestep	1 fs

### Interatomic potential functions

In MD simulations, the potential function plays a decisive role in the simulation results. The properties of the material are fundamentally controlled by the interaction between atoms. In this research, the interatomic potentials between workpiece atoms and tool atoms are Morse potential, EAM potential, and Tersoff potential. The interaction between Cu atoms and C atoms is calculated by Morse potential which is shown as Eq. (1).

1 $$ u\left({r}_{ij}\right)=D\left[ \exp \left(-2\alpha \left({r}_{ij}-{r}_0\right)\right)-2 \exp \left(-\alpha \left({r}_{ij}-{r}_0\right)\right)\right] $$

where *r*_0_, *α*, and *D* are atomic spacing, elasticity modulus, and binding energy, respectively. The values of them are shown as Table 2.

**Table 2:** Parameters value in Morse potential

*r*_0_ (Ả)	α (Ả^−1^)	*D *(eV)
2.2	1.7	0.1

The interaction among Cu atoms is given by EAM potential which is shown as Eqs. (2)–(3).

2 $$ E={\displaystyle \sum_i^N\left[F\left({\rho}_i\right)+{\displaystyle \sum_{j>i}^Nu\left({r}_{ij}\right)}\right]} $$

3 $$ {\rho}_i={\displaystyle \sum_jf\left({r}_{ij}\right)} $$

The interaction between carbon atoms is calculated by Tersoff potential which is shown as Eqs. (4)–(5).

4 $$ E=\frac{1}{2}{\displaystyle \sum_{i\ne j}{V}_{ij}} $$

5 $$ {V}_{ij}={f}_c\left({r}_{ij}\right)\left[{V}_R^{\hbox{'}}\left({r}_{ij}\right)+{b}_{ij}{V}_A\left({r}_{ij}\right)\right] $$

where, *f*_*c*_(*r*_*ij*_) is truncation function between atoms, *f*_*A*_(*r*_*ij*_) is the dual potential of absorption term, *f*_*R*_(*r*_*ij*_) is the dual potential of repulsion term, *r*_*ij*_ is atomic distance between atom *i* and atom *j*.

### Analysis methods

In order to analyze the subsurface deformation and dislocation evolution and measure the depth of the subsurface deformed layer of the workpiece, the CSP is introduced, which is given by Eq. (6) [16]:

6 $$ CSP={\displaystyle \sum_{i=1,6}{\left|{R}_i+{R}_{i+6}\right|}^2} $$

where, *R*_*i*_ is the neighbor atoms with the same length, *R*_*i+6*_ is the neighbor atoms with opposite direction. The CSP values of FCC crystal, partial dislocation, stacking fault, and surface atoms are 0, 2.1, 8.3, and 24.9, respectively. The range of CSP value for typical crystal structure and atomic coloring is shown in Table 3.

**Table 3:** The range of CSP values for typical crystal structure.

_Crystal structure_	Range of CSP value	Atomic coloring
Ideal FCC	CSP ≤ 3	Default
Stacking fault	3 < CSP ≤ 7	Red
Partial dislocation	7 < CSP ≤ 9	Orange
Surface atoms	9 < CSP ≤ 20	Yellow
Surface defect atoms	CSP > 20	Green

## Results and discussion

### Subsurface defect nucleation and evolution

**Fig. 2:**
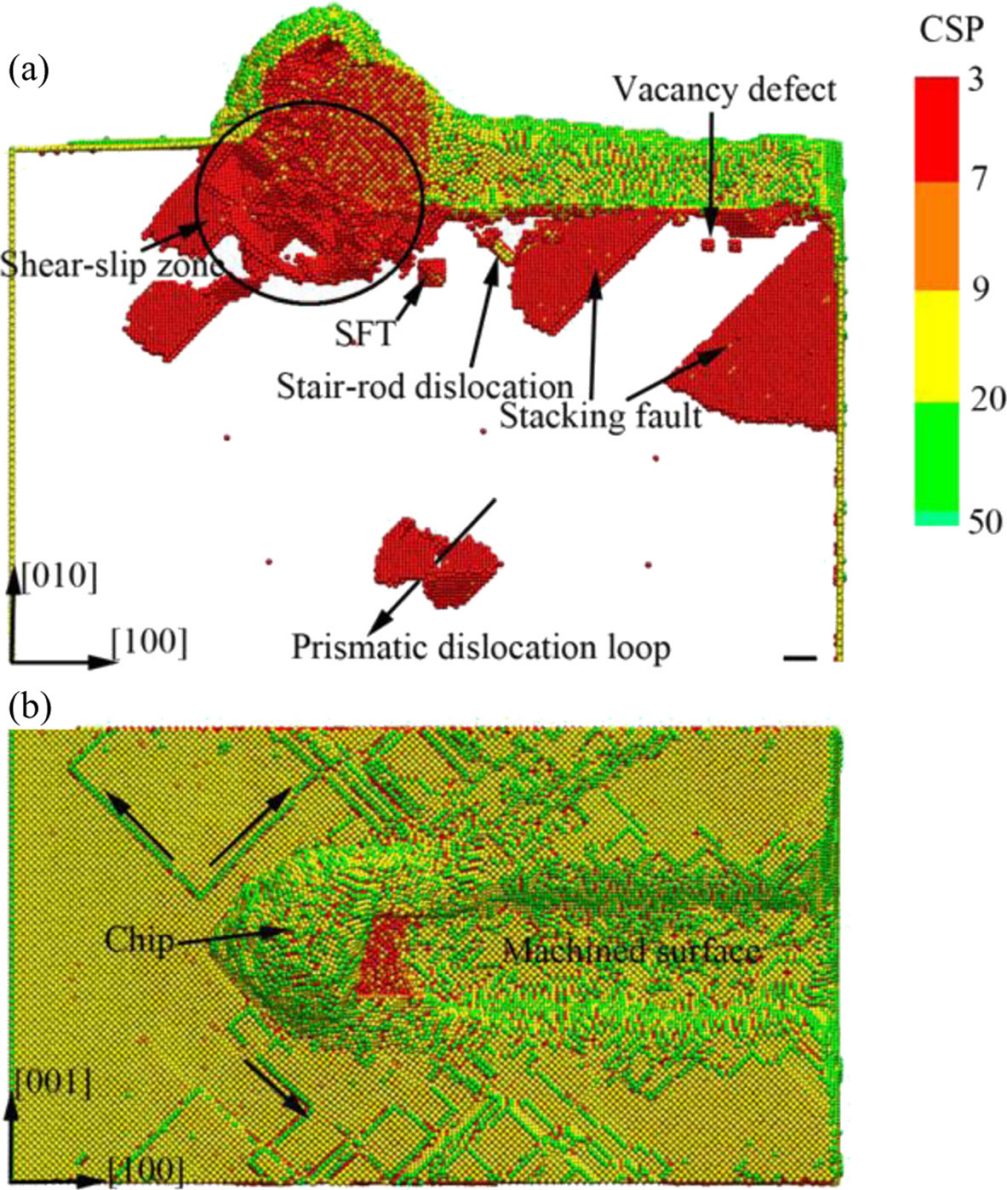
Surface and subsurface defect distribution of the workpiece in nano-cutting (color online, scale bars 5 nm). **a** Front view of the workpiece. **b** Top view of the workpiece

In nano-cutting process, with the removal of the workpiece material and the formation of the machined surface, the machined defects and residual stress are remained in the workpiece, which result in the formation of the SSD layers. To investigate the complex dislocation defect nucleation and evolution taking place in SSD layers of the workpiece, the CSP method is adopted to analyze the MD simulation result. Figure 2 is the snapshot of surface and subsurface defect distribution, in which the atoms are colored by their CSP values corresponding to Table 3. It can be seen from Fig. 2a that, under the extrusion and shearing action of cutting tool, plenty of dislocations and defects nucleated in the subsurface of the workpiece. Under the extrusion of cutting tool, the dislocation nucleation and emission of the workpiece occurred in shear-slip zone. The dislocation motion and the cutting tool moving forward cause atomic migration. The complex dislocation motion and atomic migration result in the formation of cutting chip and rough machined surface. Due to the interatomic energy transmission and assemble, dislocation nucleation of atoms also occurred at the area far away from the shear-slip zone. A prismatic dislocation loop nucleates in the subsurface of the workpiece during the early stage of the nano-cutting process, which is shown in Fig. 2a. Some atoms in stacking faults, which possess enough energy, are capable of extending to the surface of workpiece. As a result, some dislocation lines are formed at the surface, as illustrated in Fig. 2b, wherein the dislocation lines are along with [−101], [101], and [1] directions. For the atoms located under the machined surface, the dislocations annihilate with releasing of the atomic energy which has been prior stored in the dislocations formed during the nano-cutting process. Due to the atomic migration, vacancy defects are formed in the location of dislocation nucleation, while the point defects appear at the location of the dislocation annihilation. The synthetic effect of vacancy and point defects could lead to the formation of some novel and complex defect structures. For instance, stacking fault tetrahedron (SFT) [17], stair-rod dislocation [18], V shape dislocation loop [19], and stacking faults are nucleated in the subsurface of the workpiece. These defects with stable crystal structure do not disappear after the nano-machining. Moreover, the existence of those defects results in the formation of subsurface imperfect structure in which the residual stress is stockpiled. Finally, the subsurface deformed layers are formed.

**Fig. 3:**
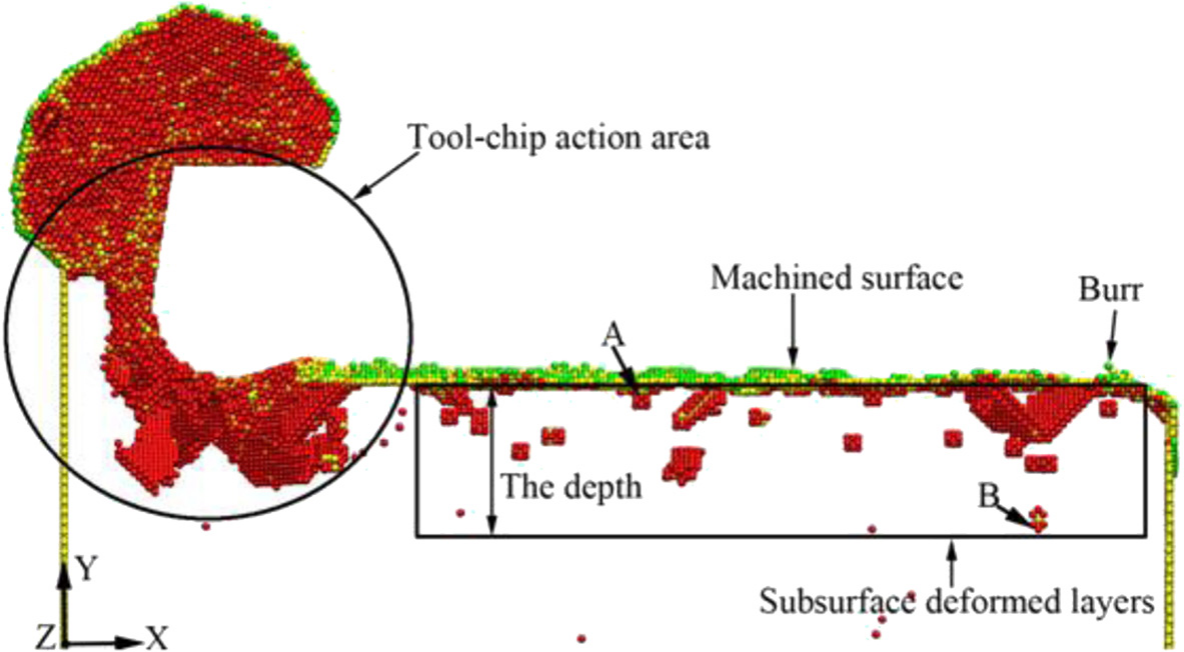
The measurement schematic diagram of the SSD layers’ depth

According to the previous discussion, the subsurface deformed layers are identified by using the CSP method. From the atomic details information, the atomic coordinate values of the atoms on the top layer and the bottom layer can be obtained. The deducting value of the two coordinate values is regarded as the depth of the SSD layers. Figure 3 is the cross-section view of the workpiece along Z direction and the schematic diagram which indicate the method to measure the depth of the SSD layers. The cut depth of Fig. 3 is 4 nm and the cut distance is 35 nm. There exists a plant of crystal defect structures in the black frames of Fig. 3, so it is identified as SSD layers. The height of the black frames is equivalent to the depth of SSD layers. In Fig. 3, atom A lies in the top layer of the black frames and atom B lies in the bottom layer of the black frames. The detailed information of atoms A and B is listed in Table 4. Therefore, the depth of SSD layers is 3.9 nm.

**Table 4:** atomic details information list

Atomic information	Atom A	Atom B
Index	103669	104274
Type	3	3
X	292.597	352.320
Y	253.403	214.572
Z	1115.779	126.835

### Effect of the cutting distance on the depth of SSD layers

**Fig. 4:**
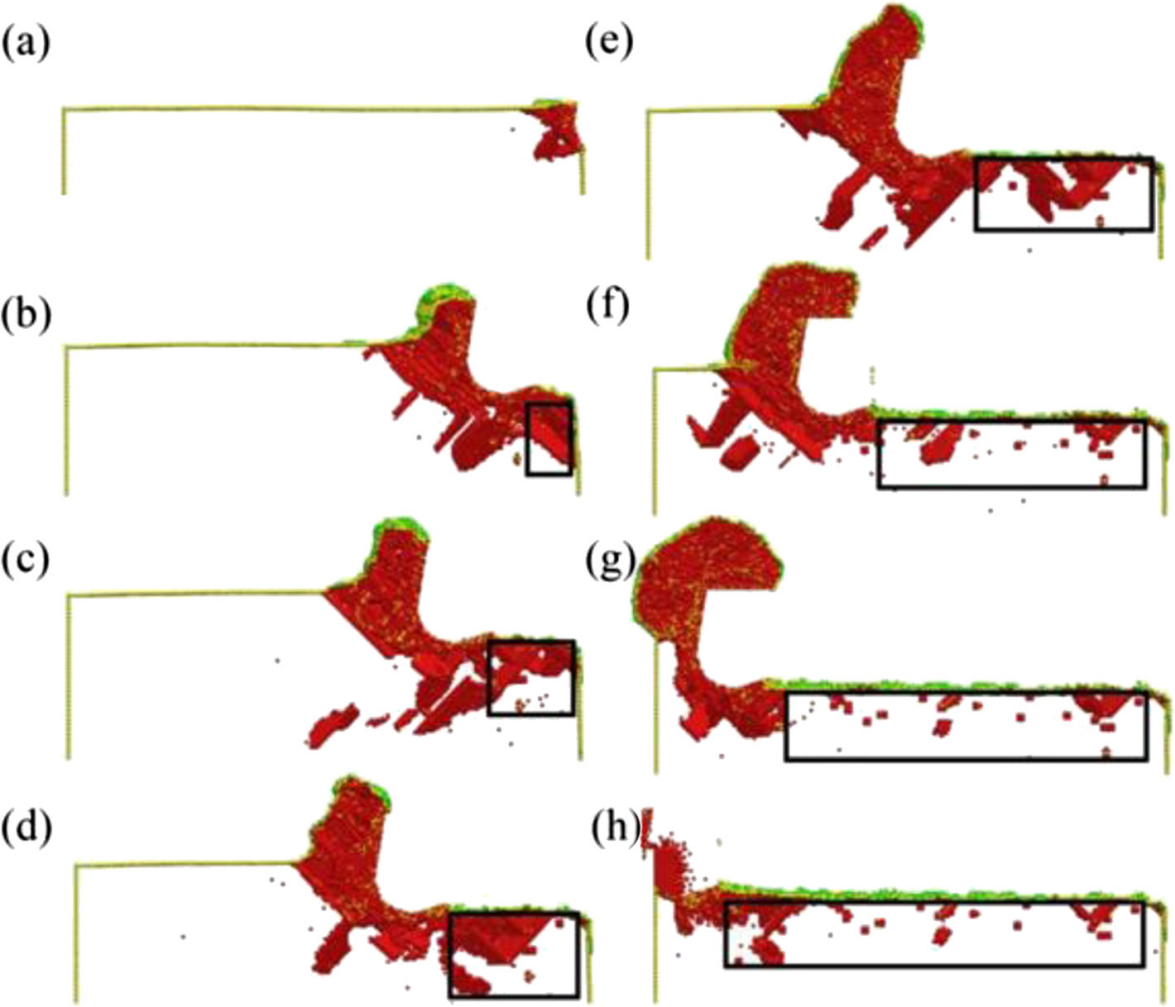
The subsurface defect distribution and the depth of the SSD layers with different cut distances

**Fig. 5:**
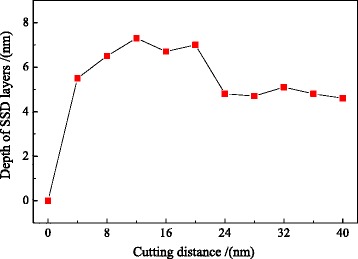
The variation of depth of the SSD layers with the cutting distance at a certain cut depth

During the nano-cutting process, the dislocation nucleation, motion, and annihilation result in the variation of the SSD layers’ depth with the cut distance changing. Therefore, it is necessary to study the effect of the cut distance on the depth of SSD layers. Figure 4 is the cross-section view of the subsurface defect distribution and the depth of SSD layers with different cutting distance at the cutting depth of 5 nm and the cutting speed of 50 m/s. The cutting distances in Fig. 4a–h, respectively, are 0, 8, 12, 16, 20, 28, 32, and 36 nm. In the early stage of the cutting process, the subsurface deformed layers are formed gradually under the extrusion and shearing action of the cutting tool. In Fig. 4, the black frames are the area of subsurface deformed layers. By using the method shown in Fig. 3, the depth of SSD layers in each snapshot is measured at the certain instantaneous moment. According to the measurement of the SSD layers’ depth, the changing curve of the SSD layers’ depth with the cutting distance changing is obtained, which is shown in Fig. 5. It can be known that the depth of SSD layers increases before the cutting distance less than 12 nm and decreases when the cutting distance is greater than 16 nm. When the cutting distance is more than 24 nm, the depth of SSD layers remains unchanged around 4.8 nm. At the early stage of the nano-cutting process, the depth of SSD layers increases significantly when the cutting distance increases to be less than 8 nm. This is because a large number of dislocations nucleated and extended at the early stage of the nano-cutting process, which result in the formation of the machined surface and the subsurface deformed layers, as shown in Fig. 4b. Therefore, the depth of SSD layers increases significantly at the beginning. In the middle stage of nano-cutting process, the dislocation internal of the workpiece continues nucleation and extension, which results in the depth of SSD layers remaining as a stable high level at the cutting distance between 8 and 20 nm, as shown in Fig. 4c–e. In the stable stage of nano-cutting process when the cutting distance is larger than 24 nm, the depth of the SSD layers decreases, which is caused by the dislocation annihilation and the defect recovery of subsurface deformed layers, as shown in Fig. 4f–h. Finally, the depth of the SSD layers keeps unchanged around 4.8 nm.

### Effect of the cutting depth on the depth of SSD layers

**Fig. 6:**
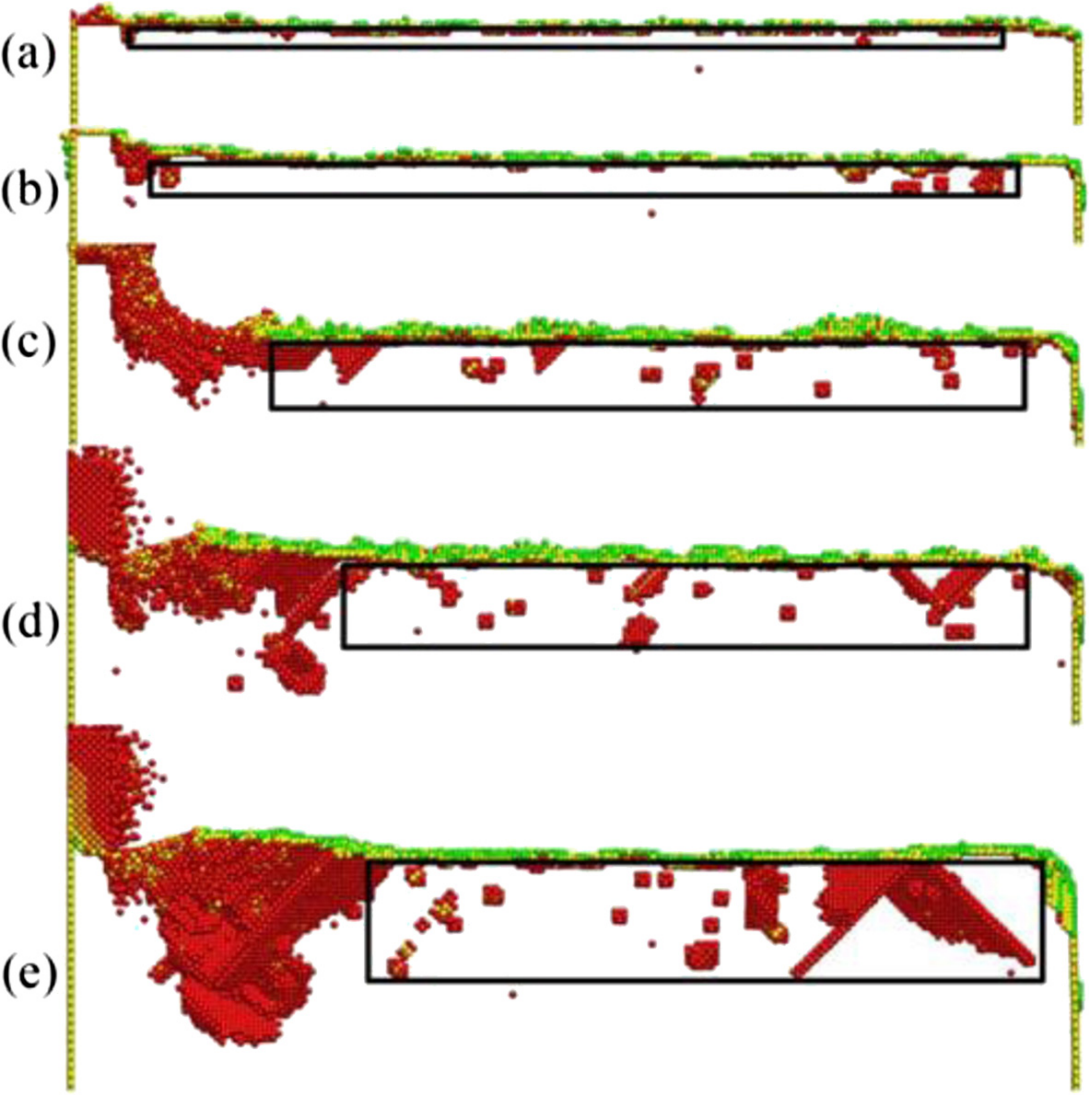
Subsurface defect distribution with different cutting depth

From the previous research, the depth of SSD layers approaches a stable value when the cutting distance is larger than a particular value. In actual nano-cutting process, because of the vibration of the ultra-precision machine tools and the cutting tool, the cut depth changes constantly. Therefore, it is necessary to study the effect of the cut depth on the depth of SSD layers. Figure 6 is the snapshot of the subsurface defect distribution with different cutting depth, in which the cutting depth values of Fig. 6a–e, respectively, are 1, 2, 3, 4, 6, and 8 nm. It can be seen from Fig. 6 that the depth of SSD layers increases with the increase of the cutting depth. The amount and type of the subsurface defects structure increase significantly with the increase of the cutting depth. It is because the increase of the back engagement of the cutting edge makes the extrusion shearing of the cutting tool action on the workpiece exacerbate, which aggravates the crystal deformation of the workpiece. The deformation results in the energy aggregation of lattice deformation enlarging, which stored in the deformed crystal lattice of the workpiece. Because of the enough energy of the subsurface atoms in the workpiece, the dislocation defects nucleated in this area extend adequately into complex defect structure, which will further affect the depth and performance of SSD layers.

**Fig. 7:**
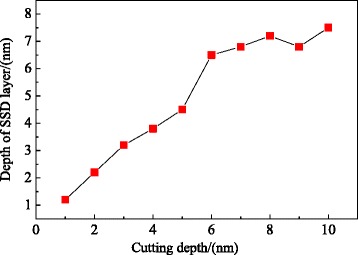
The depth of the SSD layer variation with the cutting depth

Figure 7 is the depth of the subsurface deformed layer variation with the cutting depth increase up to the speed of 100 m/s, in which the depth of SSD layers is corresponding to SSD layers’ depth during the stable stage of nano-cutting. It can be known from Fig. 7 that the depth of SSD layers in direct proportion increased with the cutting depth, when the cutting depth is less than 6 nm. When the cutting depth is greater than 6 nm, the depth of SSD layers remain unchanged at 7 nm, approximately. The cutting depth has an extremely important effect on the formation of SSD layers and the subsurface quality of the workpiece.

### Effect of the cutting speed on the depth of the SSD layers

**Fig. 8:**
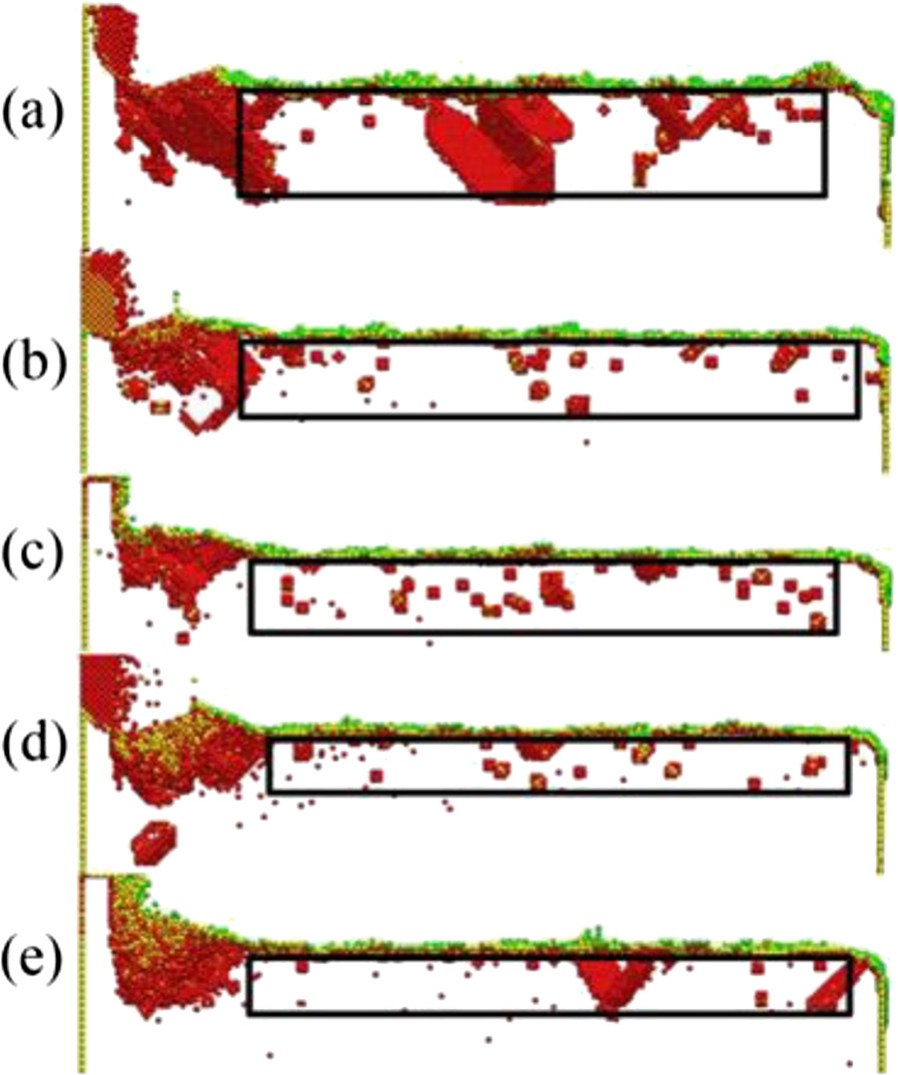
Subsurface defect distribution with different cutting speed

It is indicated in the related study of nano cutting that the cutting speed has great influence on the removal of workpiece material, the formation of chip and machined surface, and the dislocation nucleation and motion inside the workpiece, which will affect the formation process of the subsurface deformed layers and finally affect the depth of SSD layers. In order to study the influence of cutting speed on the formation of SSD layers in nano-cutting, the MD simulations of nano-cutting process with different cutting speed are performed in this research. The simulation results showed that the depth of SSD layers gradually decrease with the increase of the cutting speed. Figure 8 is the subsurface defect distribution snapshot with different cutting speeds, in which the cutting depth is 4 nm and the cutting speed of Fig. 8a–e are 50, 150, 200, 250, and 300 m/s, respectively. From Fig. 8, it can be seen that, with large amount and variety of the subsurface defects in the workpiece, the injury of subsurface is serious and the depth of the SSD layers is the largest at a cutting speed of 50 m/s for a cutting depth of 4 nm. With the cutting speed getting large, the amount and variety of subsurface defects in the workpiece decrease, and the depth of SSD layers decreases remarkably. This is because the dislocation nucleation in the workpiece occurs with sufficient extension, motion, and annihilation when the cutting speed is low, and finally, a large amount of defects in the subsurface of the workpiece remain. When the cutting speed is high, the removal of the workpiece material is complete in a short period. Therefore, the dislocation annihilation of atoms occurs before the dislocation extension and motion. Finally, there exist a few defect structures in the subsurface of the workpiece. The quality of the workpiece subsurface becomes better with the cutting speed getting larger within certain realms.

**Fig. 9:**
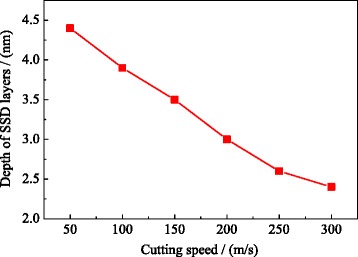
The depth of the SSD layer variation with the cutting speed

Figure 9 shows the variation of depth of SSD layers with the cutting speed. It is shown that the depth of SSD layer is 4.4 nm at the cutting speed of 50 m/s. The depth of SSD layers significantly increase with the cutting speed. When the cutting speed reaches up to 300 m/s, the depth of SSD layers is reduced to 2.4 nm. This is because the increase of the cutting speed affects the dislocation nucleation, motion, and annihilation, and finally affects the depth of SSD layers. Therefore, at a certain cutting depth, the higher the cutting speed is, the better the quality of the subsurface is.

## Conclusions

Based on the molecular dynamics simulation method and the crystal defect analysis technology, the simulation of the nano-cutting process is carried out and the distribution of subsurface defect structure is analyzed. According to the identified defect structure, three-dimensional visualization and measurement technology are used to measure the depth of the subsurface deformed layers. It can be found that the depth of SSD layer dose vary with the cutting distance, cutting depth, and cutting speed. The novel results can be summarized as follows:

1. In the process of nano-cutting, the dislocation nucleation, motion, and annihilation result in a large number of defect structures existing in the subsurface of the workpiece. For instance, stair-rod dislocations, stacking fault tetrahedron, atomic clusters, vacancy defects, and point defects are formed in the workpiece. Finally, the subsurface deformed layers are formed.
2. When the cutting depth remains constant, the depth of SSD layers increases when the cutting distance increases to be less than 8 nm, and then decreases when the cutting distance is between 8 and 24 nm. When cutting distance is greater than 24 nm, the depth of SSD layers reaches a stable value.
3. The depth of SSD layers increases proportionally with the cutting depth when the cutting depth is less than 6 nm. When the cutting depth is greater than 6 nm, the depth of SSD layers remain unchanged at about 7 nm.
4. The depth of SSD layers significantly decreases with the increase in cutting speed. The quality of the workpiece subsurface becomes better with the cutting speed getting larger within certain realms.
